# The Steady Growth of the Voluntary System

**Published:** 1919-12-20

**Authors:** 


### The Hospital

The Workers' Newspaper of Administrative Medicine and Institutional Life, Administration, National Insurance and Health.

No. 1750, Vol. LXVII. SATURDAY, DECEMBER 20, 1919. PRICE TWOPENCE

### THE STEADY GROWTH OF THE VOLUNTARY SYSTEM.

Our issue to-day may be regarded as the most remarkable number we have had the privilege to prepare for public circulation since The Hospital was first published in October 1886. It is remarkable because it deals mainly with the voluntary system of hospital support, and contains a mass of interesting information, not only concerning the origin and growth of King Edward's Hospital Fund, but also the signed statement of 100 or more Chairmen of voluntary hospitals situated in Great Britain, and a scheme whereby the voluntary system can be readily placed on a sound financial basis, and acquire guarantees of permanence and stability, impossible of attainment, previously.

The twenty-second annual meeting of the Council of King Edward's Hospital Fund was held on the 16th inst., at which the Prince of Wales, the President of the Fund, read a letter from His Majesty King George commending the voluntary system to the support of the ever-generous public, that they may make it possible for the voluntary hospitals to keep abreast of the times in their great mission of healing and mercy. It says much for the success of this Fund after twenty-one years' continuous existence that his Majesty should express his "gratitude to the Chairmen, Secretaries, and members of the various Committees for their valuable, untiring, and devoted services."

The Prince of Wales, who all were sorry, though not surprised, to see was suffering from a severe cold, made an enthusiastic and admirable Chairman, as he always does. He made a memorable apology to the Council for addressing them through a written speech, which he declared he did with great personal reluctance, owing to the fact that the pressure of engagements upon him since he returned from Canada and the United States had been so great, that he could not find time to prepare the notes for a speech to that meeting, but his will was to make none but his own speech to the members of his Council from that day forward. His Royal Highness displayed gratifying signs of the effects of his arduous efforts and unceasing exertions since the Armistice, whilst discharging the duties of Chairman with admirable ability and attractive earnestness of purpose. The occasion was memorable, seeing that the sum to be distributed, £230,000, was exceptional, against £57,000 in its first year, 1897, and £150,000, the sum regarded at the outset as the goal at which to aim under King George's presidency. £230,000 was an exceptionally large increase, and they would not, of course, expect to maintain so great a sum in another year, unless the Fund was placed, by the liberality of the public, in a position to do so. Full details of the development of the Fund, and the continuous gift of large sums to it, will be found dealt with on pages 261 to 266.

When the Fund was started, like all great movements, there were not wanting detractors who prophesied failure, but King Edward VII. had his heart in the Fund, as his son and grandson have theirs. Its administration from the commencement has been probably as near perfection as human affairs can be, and to-day the Fund has won for itself a position of confidence in the hearts of all classes of the people. Indeed the utility and supreme value of its work and influence are progressively manifested year by year.

The Voluntary Hospitals have rendered splendid service to the people of these islands, the nation and the Empire during the war. Those amongst our warriors and women workers who have had occasion to occupy a bed in any one of these institutions have become warmly impressed with their value; the strength of purpose which animates everyone connected with them, who puts the claims of the patient before all other considerations, and makes these institutions ideal residences for the sick, in direct proportion to the severity of their needs and the necessity for the very highest medical, surgical and nursing care which the world contains. The war has demonstrated that the claims here put forward are not only justified, but are under rather than over stated. We have no doubt, that whereas the Voluntary Hospitals in the past have been supported by gifts and personal service from only about one in ten of the population, resident in the district which each may serve, the declaration of the Armistice, and the settling down of all classes of the community on the conclusion of Peace, will bring the number of those who make a point of continuously rendering help to the Voluntary Hospitals, to not less than one in two of the population, against one in 100 twenty-two years ago, and one in ten at the present time. It will be an added strength to the nation, and a welcome guarantee to every hospital patient, and all who take a personal interest in the welfare of these institutions, that at least one-half of the whole population of Great Britain are convinced and active supporters of the Voluntary Hospitals, the efficiency of which is the envy of our neighbours and the pride of all hospital workers.

H.R.H. the Prince of Wales' presence at the twenty-second annual meeting of the Council will be remembered for the interest and whole-hearted attention which he brought to the discharge of the duties of his office. It will be a splendid recognition by the nation of their appreciation of the late King Edward's heartfelt and successful efforts, to make the King's Hospital Fund supremely successful. Should the further development suggested to the nation in the *Times* of the 17th inst. commend itself to the hospital chairmen and their supporters throughout Great Britain, and be adopted, then the voluntary system of hospitals, which is admittedly the best in the world, must prove an asset of supreme importance not only to the nation but to the Empire.

